# In Vitro Cytotoxicity Evaluation of the Magnéli Phase Titanium Suboxides (Ti_x_O_2x−1_) on A549 Human Lung Cells

**DOI:** 10.3390/ijms20010196

**Published:** 2019-01-08

**Authors:** Veno Kononenko, Damjana Drobne

**Affiliations:** Department of Biology, Biotechnical Faculty, University of Ljubljana, Večna pot 111, 1000 Ljubljana, Slovenia; veno.kononenko@bf.uni-lj.si

**Keywords:** A549 cells, nanoparticles, cytotoxicity, nanotoxicity, ROS, calcium ions, lysosomes

## Abstract

The use of titanium suboxides, known as Magnéli phase TiO_x_, is expected to increase in the near future due to their desirable properties. In order to use Magnéli phase TiO_x_ nanoparticles safely, it is necessary to know how nanoparticles interact with biological systems. In this study, the cytotoxicity of three different Magnéli TiO_x_ nanoparticles was evaluated using human lung A549 cells and the results were compared with hazard data on two different TiO_2_ nanoparticles whose biological interactions have already been extensively studied. After A549 cells were exposed to nanoparticles, the metabolic activity was measured by the Resazurin assay, the amount of cellular proteins was measured by the Coomassie Blue assay, and lysosomal integrity was measured by the Neutral Red Uptake assay. In order to investigate possible modes of particle actions, intracellular Ca^2+^ level, reactive oxygen species (ROS) production, and photo-oxidative disruptions of lysosomal membranes were assessed. All experiments were performed in serum-containing and in serum-deprived cell culture mediums. In addition, the photocatalytic activity of Magnéli TiO_x_ and TiO_2_ nanoparticles was measured. The results show that Magnéli TiO_x_ nanoparticles increase intracellular Ca^2+^ but not ROS levels. In contrast, TiO_2_ nanoparticles increase ROS levels, resulting in a higher cytotoxicity. Although Magnéli TiO_x_ nanoparticles showed a lower UV-A photocatalytic activity, the photo-stability of the lysosomal membranes was decreased by a greater extent, possibly due to particle accumulation inside lysosomes. We provide evidence that Magnéli TiO_x_ nanoparticles have lower overall biological activity when compared with the two TiO_2_ formulations. However, some unique cellular interactions were detected and should be further studied in line with possible Magnéli TiO_x_ application. We conclude that Magnéli phase nanoparticles could be considered as low toxic material same as other forms of titanium oxide particles.

## 1. Introduction

Nanoparticles (NPs) are nowadays produced in large quantities and used in a variety of applications, making human exposure to NPs inevitable. NPs can enter the body through inhalation, ingestion, or injections, where NPs come in contact with cells. This leads to versatile NP-cell interactions and a variety of cellular responses [1]. Numerous investigations have already been conducted that studied cell interactions with various types of NPs, from polymeric NPs [2], lipid-based NPs [3], carbon based NPs [4,5,6], and metal oxide NPs [7,8,9]. Nevertheless, numerous NPs with modified or new properties are being developed every day, and understanding their interactions with cells and cellular responses is an important scientific issue that has to be addressed in order to assure their safe and efficient use [10].

Sub-stoichiometric titanium oxides with the general formula Ti_n_O_2n−1_ (with *n* values from 4 to 10), known as Magnéli phase TiO_x_, have recently attracted much attention because of their desirable properties, such as high electrical conductivity and oxidation-resistant properties [11,12]. Magnéli phase TiO_x_ can be used to form electrodes in a variety of electrochemical processes, in battery and fuel cell applications, and in environmental technologies for the oxidation of organic wastes in water and soil remediation [13]. The most electrically conductive Magnéli phase titanium sub-oxide is Ti_4_O_7_, whose conductivity is comparable to graphite (1000 S/cm) [14]. Magnéli phase TiO_x_ can be produced from titanium dioxide (TiO_2_), a well-studied and widely used material [14]. Bulk TiO_2_ is suitable for numerous commercial applications including its use as a white pigment in paints, papers, and plastics, as well as other uses in cosmetics, medicine, and in the food industry. The nanostructured forms of TiO_2_ have several desirable properties that can be used in sensors, solar cells, medical implants, and photodynamic therapy [15,16,17,18,19,20,21]. TiO_2_ is a semiconductor, but its electrical conductivity is enhanced when it is converted to sub-stoichiometric Magnéli phase TiO_x_ [22].

From a toxicological perspective, TiO_2_ NPs are one of the most—if not the most—extensively studied metal oxide NP, but interestingly there are only a few studies evaluating the safety of Magnéli phase TiO_x_ NPs [23,24], and none of them investigated the possible mechanisms of TiO_x_ toxicity. Increasing synthesis and usage of Magnéli phase TiO_x_ necessitates its hazard evaluation. In addition to increasing intentional production of Magnéli phase TiO_x_, there is also recent evidence that large quantities of Magnéli phase TiO_x_ are being produced and released into the environment unintentionally by the coal-burning industry [23]. Because of this, a comprehensive Magnéli phase TiO_x_ NP toxicity evaluation using several environmentally relevant organisms from different trophic levels was recently produced [24]. Nevertheless, there is still a large gap in our understanding regarding the mechanisms driving the observed effects post Magnéli NP exposure. Numerous mechanisms may be responsible for the adverse effect of NPs [25,26]. The toxicity of NPs is most frequently attributed to oxidative stress, leading to the damage of biomolecules and cell organelles. NPs that come into contact with cells can be endocytosed, entering endo-lysosomal compartments of the cells, which can lead to lysosomal dysfunction with potential functional consequences [9,27]. Toxicity of NPs is often accompanied by the perturbation of intracellular Ca^2+^ homeostasis, which is associated with energetic and metabolic imbalance and other cellular dysfunctions [26,28,29].

In this study, the cytotoxicity of three different Magnéli phase TiO_x_ NPs was evaluated. For comparison, all Magnéli phase experiments were performed in parallel with two different TiO_2_ NPs with similar hydrodynamic diameter size ranges. Human lung A549 cells were used as an in vitro cell model. These cells are well characterized and widely used in nanotoxicological studies, since the lung represents an important entry route for unintentionally inhaled or intentionally lung administered NPs. In all experiments, we used A549 cells under normal cell culturing conditions (cells treated with NPs in fully supplemented cell culture medium) and under starving conditions (cells treated with NPs in serum-deprived cell culture medium). The effects of NPs were evaluated by different assays in order to evaluate different endpoints. Intracellular reactive oxygen species (ROS) production was monitored by oxidation-sensitive fluorescent dye DCFH-DA and flow cytometry. Lysosomal stability and the photo-oxidative disruption of lysosomal membranes was evaluated by Acridine Orange (AO) relocalization assay. The intracellular Ca^2+^ level was monitored by the use of Ca^2+^-sensitive fluorescent dye Fluo-4 and microscopic observation. In addition, the photocatalytic activity of Magnéli phase TiO_x_ and TiO_2_ NPs was assessed through UV-A photocatalytic bleaching of methylene blue dye. Such a study is important because of the expectation that the use of Magnéli phase TiO_x_ NPs will increase in the near future and the consequences of their interactions with cells are still largely unknown. The aim of this study was to evaluate the hazard potential of Magnéli phase TiO_x_ and to provide this information to producers, users, and regulators.

## 2. Results

### 2.1. Nanoparticle Characteristics

**Figure 1:**
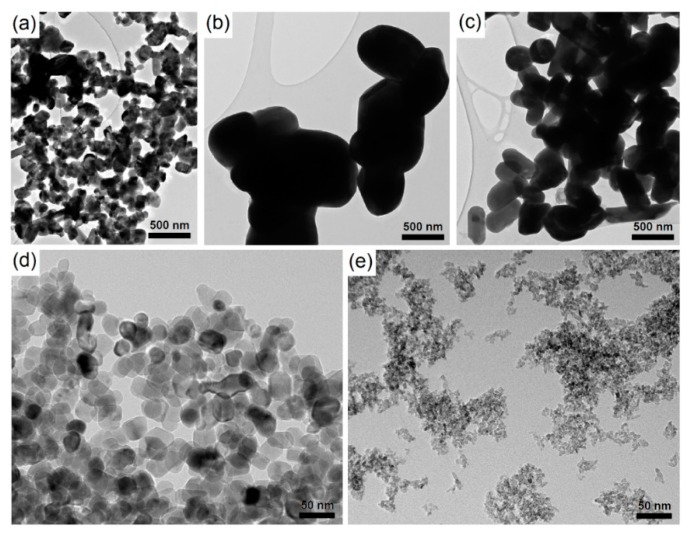
Transmission electron microscopy images of (**a**) Magnéli-A nanoparticles, (**b**) Magnéli-B nanoparticles, (**c**) Magnéli-C nanoparticles, (**d**) TiO_2_-A nanoparticles, and (**e**) TiO_2_-B nanoparticles.

Three different Magnéli phase TiO_x_ NPs and two different TiO_2_ NPs were used in this study (Figure 1). The average diameter (determined by TEM analysis), hydrodynamic diameter (determined with dynamic light scattering [DLS]), zeta potential, and UV-VIS absorbance maximum for all used NPs are presented in Table 1. Results of photocatalytic bleaching of methylene blue showed the highest UV-A photocatalytic activity for TiO_2_-B NPs, followed by TiO_2_-A NPs. All used Magnéli phase TiO_x_ NPs were also photoactive under the influence of UV-A light, but to a lower extent than the TiO_2_ NPs (Figure S1). The UV-VIS absorbance spectrum for all used NPs are presented in Figure S2.

### 2.2. Cellular Protein Amount Assay

**Figure 2:**
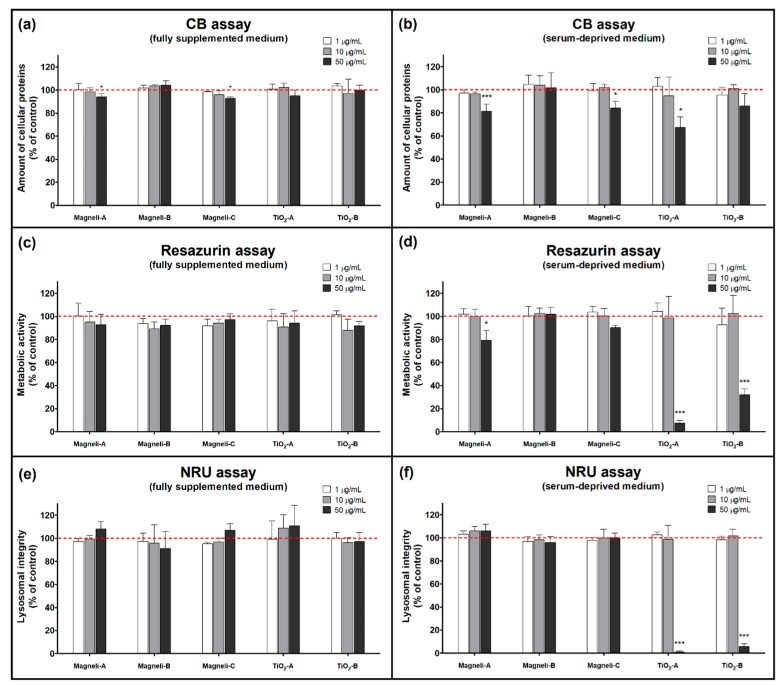
Cytotoxicity of Magnéli phase TiO_x_ and TiO_2_ nanoparticles. Cytotoxicity was assessed after 24 h exposure to A549 cells, evaluated by (**a**,**b**) Coomassie Blue (CB) assay, (**c**,**d**) Resazurin assay, and (**e**,**f**) neutral red uptake (NRU) assay. For each treatment condition, at least three independent experimental repeats, each with five replicates, were performed. Data is presented as mean percentage (+SD) of untreated controls (dashed line). Asterisk presents significant difference with respect to the untreated control cells (* equals *p* < 0.05; *** equals *p* < 0.001; ANOVA with Bonferroni’s post test).

The amount of cellular proteins, which is proportional to the cell number, was evaluated by the Coomassie Blue (CB) assay [20]. CB dye binds to all cellular proteins, making cellular proteins amount evaluation possible by spectrophotometric measurement. The CB assay revealed that none of the used NPs had a great influence on cell number in fully supplemented cell culture medium (Figure 2a). Experiments performed in serum-deprived medium revealed a small but statistically significant decrease in cell number when cells were exposed to Magnéli-A, Magnéli-C, and TiO_2_-A NPs in a dose dependent manner (Figure 2b).

### 2.3. Metabolic Activity Assay

The metabolic activity of NP exposed A549 cells was measured by Resazurin assay, utilizing a weakly fluorescent resazurin as an oxidation-reduction indicator that undergoes colorimetric change to a highly fluorescent resorufin in response to cellular metabolic reduction. In fully supplemented cell culture medium, none of the used NPs had a significant effect on metabolic activity of A549 cells (Figure 2c). In contrast, both TiO_2_ NPs at 50 µg/mL significantly decreased metabolic activity of A549 cells in serum-deprived medium. This decrease cannot be explained just by a decreased cell number, since the CB assay did not show a comparable decrease in cell number. While Magnéli-B and Magnéli-C NPs did not cause any significant decrease in metabolic activity, Magnéli-A NPs caused a small but statistically significant decrease in metabolic activity of A549 cells in serum-deprived medium (Figure 2d).

### 2.4. Lysosomal Integrity Assay

Lysosomal integrity in NP exposed A549 cells was assessed by neutral red uptake (NRU) assay [30]. This assay is based on the ability of viable cells to maintain an acidic pH inside lysosomes (an ATP-dependent process). The weak cationic neutral red dye penetrates cell membranes and concentrates in the acid environment of the lysosomes. The quantity of the neutral red dye retained in the lysosomes can be measured spectrofluorimetrically. With the NRU assay, we did not detect any significant lysosomal integrity changes in all the NP treated A549 cells in the fully supplemented cell culture medium (Figure 2e). However, we noticed a small increase in neutral red fluorescence (approximately 10%, statistically insignificant) in A549 cells exposed to Magnéli-A, Magnéli-C, and TiO_2_-A NPs in fully supplemented cell culture medium. While none of the Magnéli phase NPs caused any significant changes in neutral red fluorescence (compared to untreated control cells) in serum-deprived medium, both the tested TiO_2_ NPs caused a significant decrease in lysosomal integrity (Figure 2f).

### 2.5. Photo-Oxidative Disruption of Lysosomal Membranes

**Figure 3:**
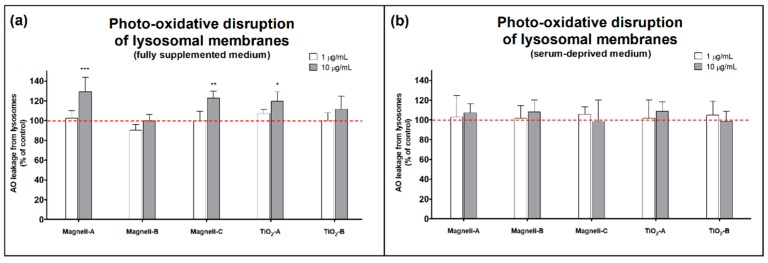
Destabilization of lysosomal membranes in nanoparticle exposed A549 cells, measured by Acridine orange (AO) leakage from lysosomes after UV-A irradiation in (**a**) fully supplemented cell medium and in (**b**) serum-deprived cell medium. For each treatment condition, at least three independent experimental repeats, each with five replicates, were performed. Results are presented as an average AO leakage (+SD) from lysosomes according to nanoparticle untreated, but UV-A irradiated controls (dashed line). An asterisk represents a significant difference with respect to the untreated control cells (* equals *p* < 0.05; ** equals *p* < 0.01; *** equals *p* < 0.001; ANOVA with Bonferroni’s post test).

To analyze the stability of lysosomal membranes and their susceptibility to oxidative disruptions, an Acridine orange (AO) relocalization assay was used [31]. AO is metachromatic fluorochrome that accumulates in the acid cell organelles, giving rise to red fluorescence upon excitation with blue light. The acidotropic AO slowly leaks into the cytosol, shifting the fluorescence to a green color. The leakage of AO is accelerated by the oxidative disruption of lysosomal membranes. By monitoring intracellular AO fluorescence before and after the cells were exposed to UV-irradiation, which triggers photo-oxidative disruptions of lysosomal membranes, the stability of lysosomal membranes can be determined. In A549 cells that were treated with Magnéli-A, Magnéli-C, and TiO_2_-A NPs in the fully supplemented cell culture medium, the UV-radiation increased the AO leakage from lysosomes (Figure 3a). In contrast, NP treatment in serum-deprived medium did not have any significant effect on AO leakage from lysosomes (Figure 3b). These NP concentration and medium composition dependent results indicate a higher destabilization of lysosomal membranes in cases where NPs are covered by serum proteins, probably due to their higher endocytosis.

### 2.6. Intracellular Ca^2+^ Level

**Figure 4:**
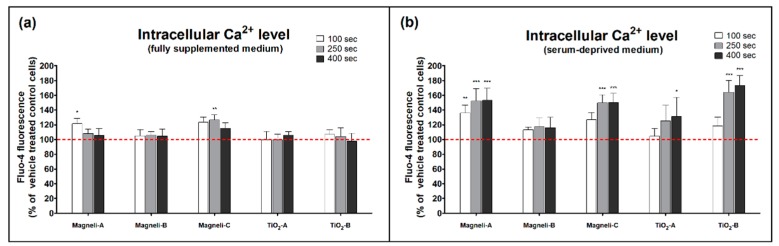
Intracellular Ca^2+^ level in A549 cells after 100, 250, and 400 s exposure to nanoparticles in (**a**) fully supplemented cell medium and in (**b**) serum-deprived cell medium. For the evaluation of the intracellular Ca^2+^ level, Ca^2+^-sensitive Fluo-4 dye was used. For each treatment condition, three independent repetitions with fluorescence of at least 20 individual cells were evaluated. Results are presented as an average Fluo-4 fluorescence (+SD) according to vehicle treated control cells (dashed line). An asterisk represents a significant difference with respect to the control cells (* equals *p* < 0.05; ** equals *p* < 0.01; *** equals *p* < 0.001; ANOVA with Bonferroni’s post test).

In order to assess the intracellular Ca^2+^ level in A549 cells after NP exposure, we used Fluo-4 AM dye and time-lapse microscopic imaging [32]. Fluo-4 is a Ca^2+^ dependent dye that exhibits a large fluorescence intensity increase upon binding with free Ca^2+^. Intracellular Ca^2+^ level in A549 cells were influenced by the addition of Magnéli-A and Magnéli-C NPs in both fully supplemented and serum-deprived cell mediums, while TiO_2_-A and TiO_2_-B NPs significantly influenced the intracellular Ca^2+^ level only in serum-deprived cell medium (Figure 4 and Figure S3). Magnéli-B did not have a significant effect on the intracellular Ca^2+^ level.

### 2.7. Intracellular Reactive Oxygen Species Levels

**Figure 5:**
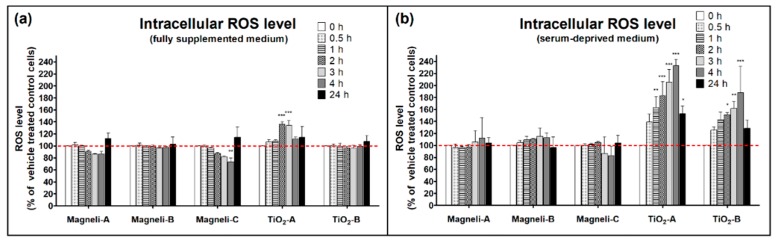
Intracellular reactive oxygen species (ROS) levels in A549 cells after 0, 0.5, 1, 2, 3, 4, and 24 h exposure to nanoparticles in (**a**) fully supplemented cell medium and in (**b**) serum-deprived cell medium. For the evaluation of intracellular ROS levels, the ROS-sensitive indicator 2′,7′-dichlorofluorescein diacetate (DCFH-DA) was used. At least 20,000 events were measured for every sample, and for each treatment condition, two repetitions were done. Results are presented as an average 2′,7′-dichlorofluorescein (DCF) fluorescence (+SD) according to vehicle treated control cells (dashed line). An asterisk presents a significant difference with respect to the control cells (* equals *p* < 0.05; ** equals *p* < 0.01; *** equals *p* < 0.001; ANOVA with Bonferroni’s post test).

The intracellular ROS levels in A549 cells exposed to all the tested titanium NPs were evaluated by non-fluorescent ROS-sensitive indicator 2′,7′-dichlorofluorescein diacetate (DCFH-DA), which is converted to the highly fluorescent 2′,7′-dichlorofluorescein (DCF) after oxidation by ROS. None of the Magnéli NPs increased intracellular ROS levels (some Magnéli NPs even slightly decreased ROS levels in comparison to vehicle treated control cells). Intracellular ROS levels in A549 cells exposed to TiO_2_ NPs were highly dependent on the cell medium and the exposure duration (Figure 5). Both TiO_2_ NPs in the serum-deprived cell medium significantly increased intracellular ROS levels, while only TiO_2_-A increased ROS levels in the fully supplemented cell medium.

## 3. Discussion

In our study, the hazard potential of the Magnéli phase titanium suboxide NPs was assessed on A549 human lung cells and compared to that of TiO_2_ NPs with the aim of providing experimental data to support the application of this new form of titanium particles. Our results showed that the cytotoxicity of all tested titanium NPs is relatively low and dependent on NP concentration and the exposure medium (Table 2). While cytotoxicity of TiO_2_ NPs occurred due to increased ROS production, we did not detect any intracellular ROS increase in cells exposed to Magnéli NPs. Cytotoxicity of Magnéli NPs was commonly accompanied by an increase in the intracellular Ca^2+^ concentration.

We showed that the effects of all tested titanium NPs is strongly dependent on the exposure medium (Table 2). Both TiO_2_ NPs significantly increased the intracellular ROS levels in serum-deprived medium, while in a fully supplemented medium only TiO_2_-A increased ROS levels, and this increase was only transient (Figure 5). It is known that cells need different growth factors found in serum for normal cell growth and proliferation, and that when serum is removed from cell medium, this by itself represents a stress for cells, making cells more sensitive to additional stresses. Another reason why the exposure medium affects the cytotoxicity of NPs is that NPs behavior is influenced by their environment. When NPs are introduced to complex biological media containing different ions, proteins, lipids, and other molecules, they are subjected to a range of forces that determine their aggregation status in a suspension. Aggregation appears to be a ubiquitous phenomenon among all NPs. Albanese and Chan (2011) [33] reported that the uptake patterns are different between single and aggregated NPs and provided evidence on a 25% decrease in uptake of aggregated Au NPs with A549 cells in comparison to single and monodisperse NPs. They highlighted the need to investigate the behavior of aggregates with cells on a case-by-case basis. Several reports have demonstrated that the presence of serum proteins in cell culture medium results in improved NP suspension stability [34]. For example, Ji et al. (2010) [35] showed that fetal bovine serum (FBS) is an effective dispersing agent for TiO_2_ nanoparticles in different media. This is in agreement with our results, which showed that all used titanium particles formed smaller aggregates in serum-free medium than in full cell medium (Table 1). In addition, it is known that aggregation of NPs significantly affects their interactions with the cells [34]. Many studies have shown that cytotoxicity of different NPs is size dependent [8,36,37,38], but not in a simple and straightforward relation between cellular effect and NP or aggregate size, but in an interplay between particle size, aggregate size distribution, and stability of aggregates in biological medium. Our results show that Magnéli-A and Magnéli-C are more cytotoxic in comparison to bigger Magnéli-C particles. Nevertheless, all used Magnéli phase NPs formed aggregates of comparable sizes with wide size distribution. In addition, around NPs, the particle biomolecular corona, composed of proteins and other molecules, is formed as soon as they enter a biological environment. The biomolecular corona highly affects particle properties, surface chemistry, their aggregation state, as well as their interactions with cells [39]. Serum components that are adsorbed to NP surfaces were found to provide some protection from the cytotoxic effect of endocytosed TiO_2_ NPs [39].

In contrast, it was only in the fully supplemented medium that we observed a significant oxidative disruption of the lysosomal membranes, shown by an increased AO leakage from lysosomes (Figure 3). We speculate that NPs in serum-containing medium enter cellular endo-lysosomal compartments to a greater extent when compared to NPs in serum-deprived medium. It has already been shown that the amount of NPs internalized by the cells is proportional to the amount of adsorbed proteins on the NPs surface [40]. It is also known that protein adsorption to the NP surface reduces the size of NP aggregates and agglomerates [39,41], and that NP agglomeration and aggregation status have a significant influence on NP endocytosis [42]. We observed that all the tested Magnéli phase NPs had a bimodal hydrodynamic size distribution when in the fully supplemented medium, in which the largest particle aggregate/agglomerate population was comparable to the unimodal size distribution value found in the same NPs when in the serum deprived medium (Table 1). The same type of NPs that disturbed the lysosomal membranes (Magnéli-A, Magnéli-C, and TiO_2_-A; Figure 3a) also increased the cellular amounts of acid organelles in fully supplemented medium (shown by the increased neutral red fluorescence; Figure 2e), which is a known consequence of loading lysosomes with non-degraded material [43]. It is known that TiO_2_ NPs exhibits high photocatalytic activity, leading to ROS photogeneration and oxidative disruption of lysosomal membranes [20,21]. Magnéli phase NPs, on the other hand, have no such photocatalytic activity (Figure S1), and the mechanism of their lysosomal disruptions remains unclear. Lysosomes are one of the main intracellular loading sites for NPs and as such are an important potential target for cellular impairment. Lysosomes participate in the digestion of endocytosed and autophagocytosed material, but are also involved in the maintenance of intracellular Ca^2+^ homeostasis [44]. NPs that enter the endo-lysosomal system can lead to lysosomal dysfunction that is associated with several diseases [45,46]. NPs can inhibit the activity of lysosomal enzymes by interacting directly with enzymes, by adsorption on enzymes, by oxidative damage of enzymes, or simply by increasing the intra-lysosomal pH. Lysosomal overloading with indigestible substances like NPs may lead to severe degenerative alterations of the cells. This indicates that supposedly inert but biopersistent nanomaterials can be harmful by interfering with normal endo-lysosomal functioning [9]. Lysosomal destabilization has already been shown as a cytotoxicity mechanism of TiO_2_ NPs in human bronchial epithelial cells and in murine fibroblast cells [47,48]. For Magnéli phase NPs, there has been no existing data about their potential interactions with lysosomes up to now.

Our results indicate that the decreased number of cells exposed to Magnéli phase NPs is accompanied by an increase in the intracellular Ca^2+^ level. These results are in agreement with the findings of Meindl et al. [28], who showed a link between increased intracellular Ca^2+^ in human neuroblastoma SH-SY5Y cells and polystyrene NP cytotoxicity. Ca^2+^ is known for its ambivalent nature—it is essential for the normal functioning of cells, but it also conveys negative signals. The Ca^2+^ modulates all important aspects of cell life, from its origin at fertilization, to its end in the process of apoptosis. Cells precisely regulate the level of intracellular Ca^2+^, which has significant effects on cellular metabolism, signal transduction, and gene expression, but if its intracellular concentration and movements are not carefully tuned, it becomes a mediator of cell distress and cell death [29,44]. Metabolic dysfunctions and other cytotoxic effects are commonly accompanied by an increase in the intracellular Ca^2+^ concentration [29]. NPs can cause elevation in intracellular Ca^2+^ levels by promoting a Ca^2+^ influx through calcium channels, releasing Ca^2+^ from intracellular stores (such as endoplasmic reticulum, Golgi apparatus, mitochondria, lysosomes), inhibiting Ca^2+^ sequestration, or blocking Ca^2+^ efflux from the cell [29,44]. NPs can damage the cells membranes, which may contribute to increased Ca^2+^ concentrations in the cytosol with the Ca^2+^ entering from the extracellular fluid or from intracellular storages. It has been shown that NPs may stimulate the opening of Ca^2+^ channels—at least partially—via an induction of oxidative stress [49]. On the other hand, it was also shown that Ca^2+^ can activate enzymes that generate increased ROS levels [29]. However, in our study, we did not find any clear connection between the increased intracellular Ca^2+^ level and subsequent increases in the intracellular ROS levels (Table 2). This may indicate an additional well-controlled cellular signal pathway in case of exposure to Magnéli phase NPs leading to modulation of cell proliferation. It is worthwhile to further explore this mechanism.

In contrast to the cellular effect of Magnéli TiO_x_ NPs that was accompanied by the increased intracellular Ca^2+^ level, the cellular effect of TiO_2_ NPs was accompanied by increased intracellular ROS levels. ROS are produced during normal cellular processes, but NPs can increase ROS production, and this is considered the primary cause of NP toxicity. Increased ROS levels in the cell can lead to severe damage of cellular macromolecules (proteins, lipids, DNA), leading to cytotoxicity [50]. Of all the tested NPs in fully supplemented medium, only TiO_2_-A significantly increased intracellular ROS levels, and this increase was only temporary (Figure 5a). In serum-deprived medium, both TiO_2_ NPs significantly increased the ROS levels in A549 cells, while all used Magnéli phase NPs had no significant effect on ROS levels. Here, we have to emphasize that ROS experiments were performed in the dark, thus avoiding the photocatalytic actions of NPs (Figure S1).

Our results show that different Magnéli phase NPs have different effects on A549 cells. In contrast to the Magnéli-A and Magnéli-C NPs, we observed that Magnéli-B NPs did not increase the intracellular Ca^2+^ level, nor did they have any other adverse effect on the cells. It is not clear why these differences occurred. All the tested Magnéli phase NPs had broad size distribution, and although Magnéli-C NPs had the highest average diameter as assessed by TEM, they formed aggregates/agglomerates of comparable hydrodynamic sizes to the other tested Magnéli NPs (Table 1). Further research is needed in order to investigate which NP properties are responsible for these differences in the biological effects among different Magnéli phase NPs.

Some potentially harmful adverse effects of Magnéli phase NPs were detected (Figure 2 and Figure 3), but they were observed at relatively high exposure concentrations (≥10 µg/mL), meaning that Magnéli phase NPs may not pose a large hazard under biologically relevant in vivo conditions. As has already been emphasized by different researchers, toxicological studies using unrealistically high experimental doses are sometimes required in mechanistic studies, but have to be interpreted with caution [51,52]. Risk of NP adverse effects is a combination of the NPs hazard potential and exposure. If exposure does not occur, there is no risk. On the other hand, relatively benign particles with a low toxicity can cause serious adverse effects if administrated at high doses [51,53]. To assess the risk of Magnéli phase NPs, data regarding exposure during whole life cycles of particles—from production and application to disposal—is needed. In order to assure the safe use of Magnéli phase NPs, additional in vitro and in vivo research is needed. It is not expected that Magnéli phase NPs would reach a high exposure concentration to pose a realistic health risk. In addition, the mode of action indicated in our study suggests even an lower toxic potential of Magnéli phase NPs than that of TiO_2_, which is among low toxic NPs [54]. Therefore, Magnéli phase NPs could be considered as a material of low hazard potential.

## 4. Materials and Methods

### 4.1. Chemicals

Fluo-4 AM was from Invitrogen (Carlsbad, CA, USA). Cell culture media and all other chemicals used in our experiments were from Sigma-Aldrich (Steinheim, Germany) unless stated otherwise.

### 4.2. Preparation and Characterization of Nanoparticle Suspensions

In our study, we used three different Magnéli phase TiO_x_ NPs and two different TiO_2_ NPs (Table 1). In all the used Magnéli TiO_x_ NPs, the percentage of highly conductive phase Ti_4_O_7_ was higher than 70%. All used TiO_2_ NPs were in the anatase phase.

Stock suspensions of NPs at 10 mg/mL were prepared in deionized water (MilliQ, Millipore, Billerica, MA, USA [pH = 5.7, ρ = 18.5 MΩ·cm]). Before the experiments, the stock suspensions were mixed and sonicated in an ultrasonic water bath (15 min, 250 W, 50 Hz, Sonis 2GT, Iskra Pio, Šentjernej, Slovenia). Sonicated suspensions of NPs were used to prepare working NPs suspensions in the cell culture medium. The primary size of NPs in suspensions were characterized using transmission electron microscopy (TEM; JEOL 2100, Tokyo, Japan). The hydrodynamic diameter of the NPs in water, in fully supplemented cell medium, and in serum deprived cell medium (50 µg/mL) was obtained using dynamic light scattering (DLS; Analysette 12 DynaSizer, Fritsch GmbH, Idar-Oberstein, Germany). The zeta-potential of the same NP suspensions were monitored with electro-kinetic measurements of the (ZetaPALS potential analyzer, Brookhaven Instruments Corp, Holtsville, NY, USA). The UV-VIS absorption spectrum of NPs in a water suspension was measured in a UV-transparent 96-well plate by spectrophotometer (BioTek, Cytation 3, Bad Friedrichshall, Germany) from 230 to 900 nm wavelengths. The absorption of pure water at corresponding light wavelengths was subtracted from the obtained results. The photocatalytic activity of all used NPs was evaluated through the measurement of the UV-A photocatalytic bleaching of methylene blue dye according to ISO standard 10678:2010. Briefly, 10 µM methylene blue aqueous solution was mixed with 1000 µg/mL NPs and was then either UV-A light irradiated using the UV-A LED panel flood lamp (PowerMAX; PM-1600UVH/F) with an output power of 8 μW/cm^2^, or incubated in the dark. Degradation of methylene blue dye because of photocatalytic activity of used NPs was monitored every 30 min for 4 h by measuring the methylene blue light absorption at 664 nm.

### 4.3. Cell Culture

A549 cells were cultured in Dulbecco’s modified Eagle’s medium (DMEM), supplemented with 2 mM L-glutamine and 10 % (*v/v*) FBS. Cells were grown at 37 °C in a humidified atmosphere with 5% CO_2_ and were routinely passaged twice a week. A549 cells were confirmed to be Mycoplasma negative using the MycoAlert^™^ Kit (Lonza, Basel, Switzerland) and following the manufacturer’s protocol.

### 4.4. Coomassie Blue Assay

A549 cells were seeded into transparent 96-well plates at the seeding density of 2.2 × 10^4^/cm^2^ and incubated for 24 h followed by a 24 h treatment with NPs prepared in fully supplemented or serum-deprived cell culture medium. As a positive control for cytotoxicity, 0.5 mM H_2_O_2_ was used. After treatment, A549 cells were stained by CB solution (0.05% Coomassie Brilliant Blue G250 in 30% methanol, 10% acetic acid, 60% MilliQ water) for 40 min at room temperature. After rinsing with Dulbecco’s phosphate-buffered saline (DPBS), 0.1 M NaOH was added to solubilize the dye. The absorbance of CB was measured at 630 nm using a microplate reader (BioTek, Cytation 3). For each treatment condition, at least three independent experimental repeats, each with five replicates, were performed.

### 4.5. Resazurin Assay

A549 cells were seeded into black 96-well plates (2.2 × 10^4^ cells/cm^2^) and 24 h incubated for cell attachment, which was followed by a 24 h incubation with NP suspensions prepared in fully supplemented or serum-deprived cell culture medium (final concentrations of NPs was 1, 10, 50 µg/mL). As a positive control for cytotoxicity, 0.5 mM H_2_O_2_ was used. After treatment, 25 µg/mL resazurin was added to each well and incubated at 37 °C for 3 h. Fluorescence intensity of formed resorufin was measured (560/590 nm ex/em) using a spectrofluorimeter (BioTek, Cytation 3). For each treatment condition, at least three independent experimental repeats, each with five replicates, were performed.

### 4.6. Neutral Red Uptake Assay

Briefly, 2.2 × 10^4^ A549 cells/cm^2^ were seeded into black 96-well plates and incubated for 24 h to allow for cell attachment followed by a 24 h treatment with NP suspensions prepared in fully supplemented or serum-deprived cell culture medium (final concentrations of NPs was 1, 10, 50 µg/mL). As a positive control for cytotoxicity, 0.5 mM H_2_O_2_ was used. After treatment, 0.04 mg/mL neutral red dye was added to each well and cells were incubated for 2 h, allowing dye to become trapped inside the acid organelles. Cells were rinsed with DPBS, followed by releasing the internalized dye by a prepared solvent (50% *v/v* ethanol, 1% *v/v* acetic acid, and 49% *v/v* deionized water). Fluorescence of the released neutral red dye was measured spectrofluorimetrically (BioTek, Cytation 3) at the excitation wavelength of 530 nm and emission wavelength of 645 nm. For each treatment condition, at least three independent experimental repeats, each with five replicates, were performed.

### 4.7. Acridine Orange Relocalization Assay

A549 cells were seeded into black 96-well plates (2.2 × 10^4^ cells/cm^2^) and incubated for 24 h to allow for cell attachment, which was followed by a 24 h treatment with NP suspensions prepared in fully supplemented or serum-deprived cell culture medium (final concentrations of NPs were 1 and 10 µg/mL). As a positive control for lysosomal instability, 25 µM chloroquine was used. After treatment, cells were stained with 10 µg/mL AO for 15 min and washed with DPBS. Red (584 ex/612 em nm) and green (485 ex/520 em nm) AO fluorescence were measured spectrofluorimetrically (BioTek, Cytation 3) before and after cells were irradiated for 10 min by UV-A light using the UV-A LED panel flood lamp (PowerMAX; PM-1600UVH/F) with an output power of 8 μW/cm^2^. For each treatment condition, at least three independent experimental repeats, each with five replicates, were performed. Results are presented as an average increase in green fluorescence due to AO leakage from lysosomes into the cytosol.

### 4.8. Reactive Oxygen Species Measurement

A549 cells were incubated with 20 µM DCFH-DA for 30 min to allow for the loading of the ROS indicator inside the cells. Cells were rinsed with DPBS to remove unloaded dye, which was followed by the exposure to 50 µg/mL NP suspensions prepared in fully supplemented or serum-deprived cell culture medium. As a positive control for oxidative stress, 0.5 mM H_2_O_2_ was used. Intracellular DCF fluorescence was measured after 0, 0.5, 1, 2, 3, 4, and 24 h by flow cytometry (BD FACScalibur, BD Bioscience, Heidelberg, Germany) using blue laser (488 nm) and 530/30 bandpass filter. For every sample, at least 20,000 events were measured, and for each treatment condition, two repetitions were done. Results are presented as relative DCF fluorescence according to vehicle treated control cells.

### 4.9. Calcium Imaging

A549 cells (2.2 × 10^4^ cells/cm^2^) were seeded in 12-well plates with inserted sterile coverslips. After a 24-h incubation, allowing the cells to adhere, the cells on coverslips were stained by 2.5 µM Fluo-4 AM according to the manufacturer’s recommendations. After 30 min staining at 37 °C, cells were submerged in fresh DMEM cell medium for an additional 30 min to allow de-esterification of intracellular AM esters. Fluo-4 stained A549 cells in DMEM medium were monitored for the changes of intracellular calcium ion concentrations caused by the addition of 50 µg/mL NP suspension prepared in fully supplemented or serum-deprived cell culture medium. Time-lapse images of A549 cells were acquired at 50 s intervals using fluorescence microscopy (Axio Imager.Z1; Carl Zeiss, Jena, Germany). As a positive control for the increasing intracellular Ca^2+^ level, we used 10 µM calcium ionophore A23187. Changes in Fluo-4 fluorescence intensity in A549 cells were determined using ImageJ software (National Institutes of Health, Bethesda, MD, USA). For each treatment condition, three independent repetitions were performed where the fluorescence of at least 20 individual cells was evaluated.

### 4.10. Statistical Analysis

The data from all cytotoxicity experiments were expressed as the arithmetic mean ± standard deviation (SD) and were statistically analyzed by ANOVA with Bonferroni’s post test for multiple comparisons. A p value lower than 0.05 was considered statistically significant. All statistical analyses were performed using GraphPad Prism software (GraphPad Software, San Diego, CA, USA).

## 5. Conclusions

We conclude that Magnéli phase NPs are not hazardous material. Nevertheless, some potentially harmful adverse effects of Magnéli phase NPs were detected, presumably due to cellular internalization and biopersistence of the particles. These effects were dependent on the NPs concentration, exposure medium, and NP properties. We provide experimental evidence that the cellular effect of Magnéli phase NPs is accompanied by an increase in the intracellular Ca^2+^ level just several minutes after NP exposure. In contrast to TiO_2_ NPs, none of the Magnéli NPs increased intracellular ROS levels. To what extent the biological effect of Magnéli TiO_x_ is dependent on cell type, particle properties, NP-corona, endocytosis of particles, and their intracellular dynamics is a matter of further in vitro and in vivo research.

## Figures and Tables

**Table 1:** Physicochemical properties of the studied particles in different test media.

Particles	Crystal Phase ^1^	Average Diameter (nm)	Maximum Absorbance (nm)	Test Medium	Average Hydrodynamic Diameter (nm)	Zeta Potential (mV)
Magnéli-A	Magnéli phase	192 ± 148	350	Water	163 ± 67 and 472 ± 194	−32.9 ± 1.6
				Full cell medium	90 ± 42 and 946 ± 442	−20.8 ± 4.2
				Serum-free medium	991 ± 513	−4.4 ± 11.6
Magnéli-B	Magnéli phase	795 ± 298	545	Water	789 ± 280	−38.1 ± 1.4
				Full cell medium	124 ± 65 and 1087 ± 566	2.2 ± 5.9
				Serum-free medium	903 ± 506	−22.2 ± 4.3
Magnéli-C	Magnéli phase	507 ± 222	490	Water	543 ± 138	−40.6 ± 1.2
				Full cell medium	49 ± 8 and 1248 ± 216	−23.1 ± 4.6
				Serum-free medium	991 ± 681	−9.1 ± 6.7
TiO_2_-A	Anatase	30 ± 7	350	Water	684 ± 318	−6.9 ± 0.8
				Full cell medium	136 ± 65 and 824 ± 393	−0.5 ± 7.5
				Serum-free medium	2174 ± 1368	−10.0 ± 9.1
TiO_2_-B	Anatase	5.1 ± 1.2	250	Water	78 ± 35 and 473 ± 215	22.7 ± 1.3
				Full cell medium	68 ± 64	−7.5 ± 7.3
				Serum-free medium	1308 ± 451	−8.6 ± 3.3

^1^ Data provided by supplier. Notes: average diameter was determined by transmission electron microscopy (TEM). Average hydrodynamic diameter and zeta potential were determined in aqueous suspensions at 50 µg/mL.

**Table 2:** A summary of results obtained in the study.

Exposure Medium	Particles	Decreased Cell Number	Decreased Metabolic Activity	Affected Lysosomal Integrity	Photo-Oxidative Disruptions of Lysosomal Membranes	Increased ROS Levels	Increased Ca^2+^ Level
Fully supplemented cell medium	Magnéli-A	+	-	-	+++	-	+
	Magnéli-B	-	-	-	-	-	-
	Magnéli-C	+	-	-	++	-	++
	TiO_2_-A	-	-	-	+	+++	-
	TiO_2_-B	-	-	-	-	-	-
Serum-deprived cell medium	Magnéli-A	+++	+	-	-	-	+++
	Magnéli-B	-	-	-	-	-	-
	Magnéli-C	+	-	-	-	-	+++
	TiO_2_-A	+	+++	+++	-	+++	+
	TiO_2_-B	-	+++	+++	-	+++	+++

Colors and symbols denote the range of significant differences in comparison to the vehicle treated control cells (-: no significant difference; + equals *p* < 0.05; ++: *p* < 0.01; +++: *p* < 0.001; ANOVA with Bonferroni’s post test).

## Supplementary Materials

Supplementary materials can be found at http://www.mdpi.com/1422-0067/20/1/196/s1.

## Abbreviations

ANOVA	Analysis of variance
AO	Acridine Orange
ATP	Adenosine triphosphate
CB	Coomassie Blue
DCF	2′,7′-dichlorofluorescein
DCFH-DA	2′,7′-dichlorofluorescein diacetate
DLS	Dynamic Light Scattering
DMEM	Dulbecco’s modified Eagle’s medium
DPBS	Dulbecco’s phosphate-buffered saline
FBS	Fetal Bovine Serum
NPs	Nanoparticles
NRU	Neutral Red Uptake
ROS	Reactive Oxygen Species
TEM	Transmission Electron Microscopy
