# Supplementary material for: In Vitro Cytotoxicity Evaluation of the Magnéli Phase Titanium Suboxides (TixO2x−1) on A549 Human Lung Cells
Source: Int J Mol Sci. 2019 Jan 8;20(1):196. doi: 10.3390/ijms20010196 (PMC6337184; doi:10.3390/ijms20010196)
Supplement: Supplementary file 1 [file ijms-20-00196-s001.pdf]

SUPPLEMENTARY MATERIAL

In Vitro Cytotoxicity Evaluation of the Magnéli Phase Titanium Suboxides (Ti_xO_{2x-1}) on A549 Human Lung Cells

Veno Kononenko and Damjana Drobne *

Department of Biology, Biotechnical Faculty, University of Ljubljana, Večna pot 111, 1000 Ljubljana, Slovenia; damjana.drobne@bf.uni-lj.si; veno.kononenko@bf.uni-lj.si

* Correspondence: damjana.drobne@bf.uni-lj.si; Tel.: +386 1 3203 375

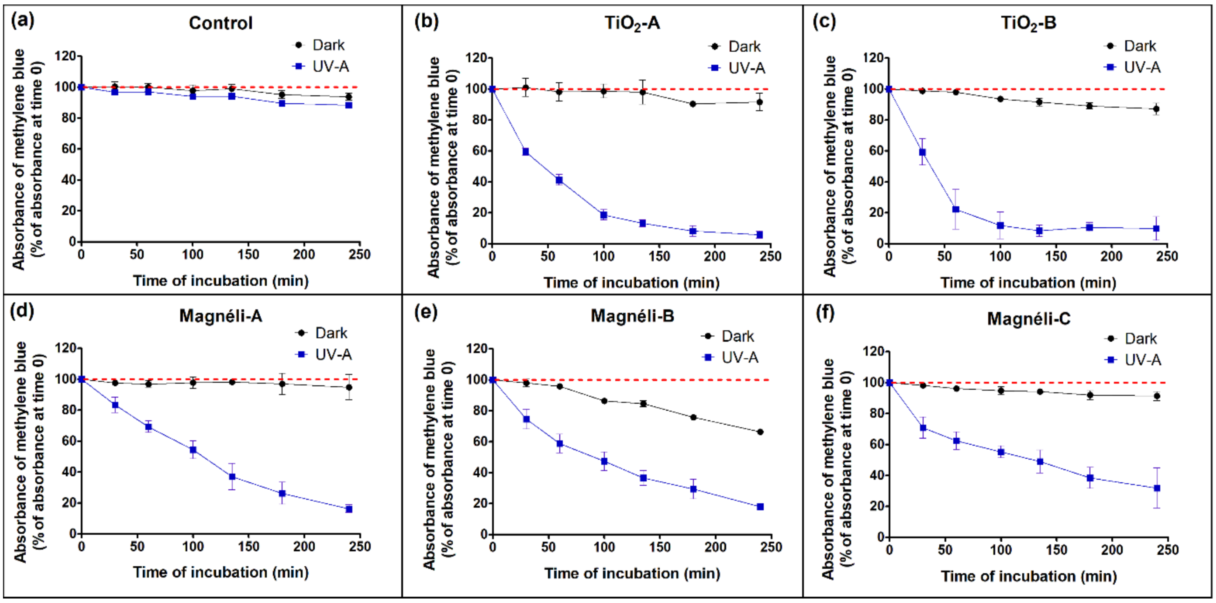

Figure S1. Photocatalytic activity of all used particles evaluated by the measurement of the UV-A photocatalytic bleaching of methylene blue dye. Bleaching of methylene blue dye in (a) control samples, (b) samples with TiO_2-A nanoparticles, (c) samples with TiO_2-B nanoparticles, (d) samples with Magnéli-A nanoparticles, (e) samples with Magnéli-B nanoparticles, and (f) samples with Magnéli-C nanoparticles.

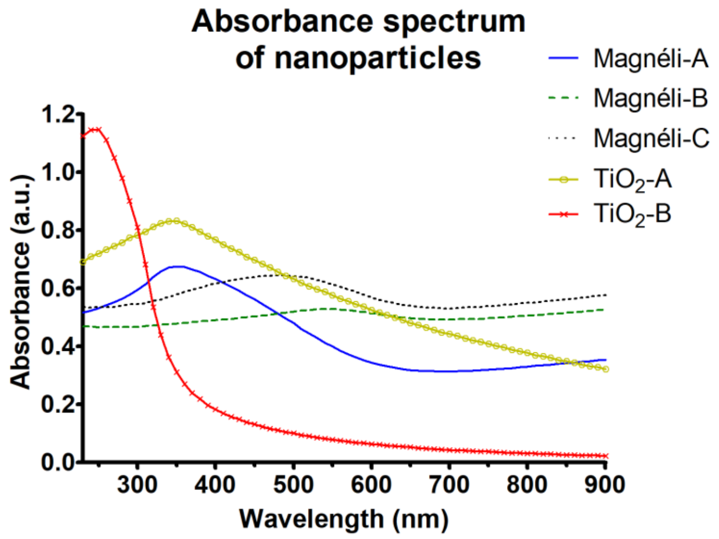

Figure S2. UV-VIS absorption spectrum of used nanoparticles (NPs).

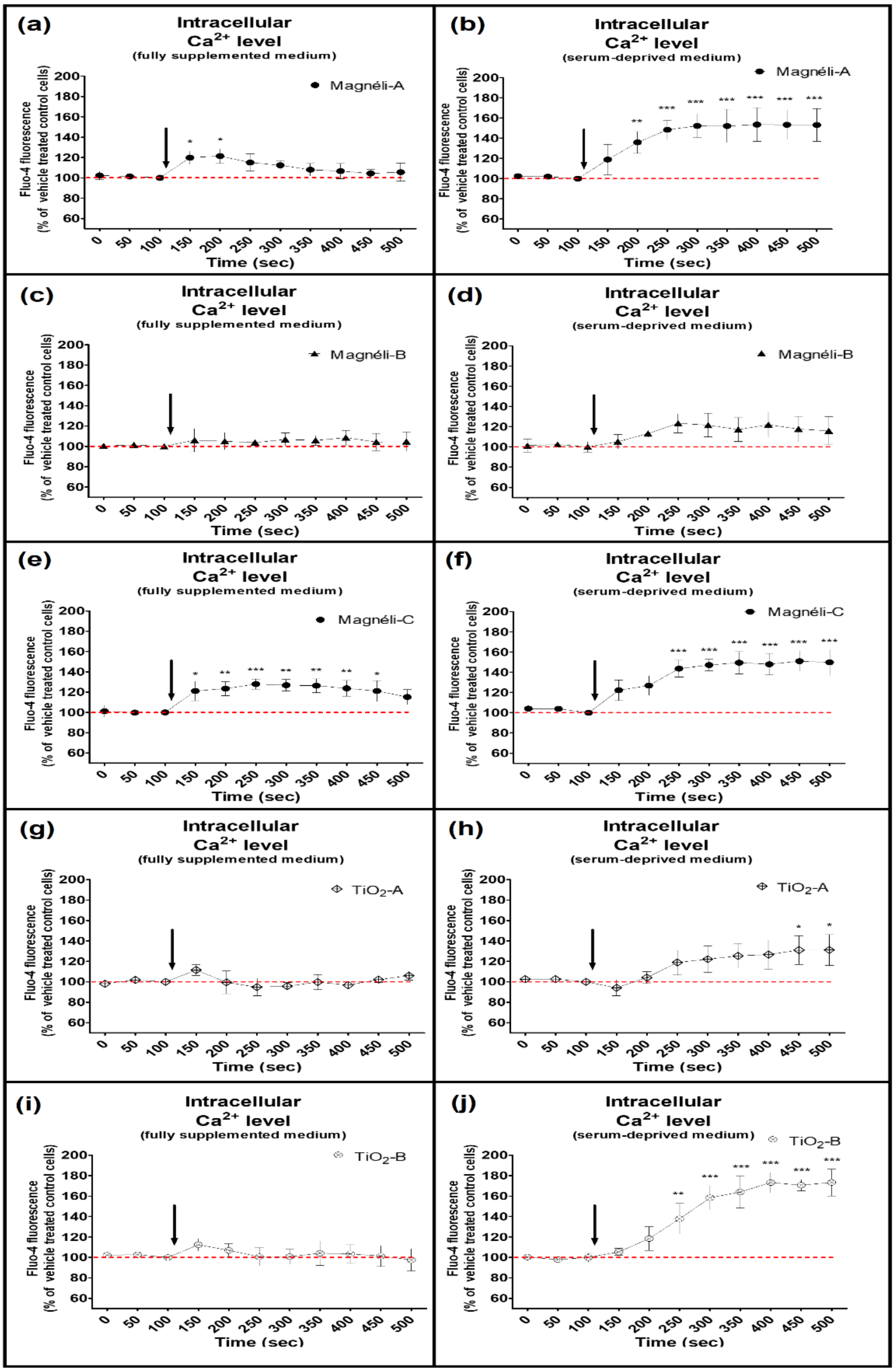

Figure S3. Intracellular Ca^{2+} level in A549 cells after exposure to (**a, b**) Magnéli-A, (**c, d**) Magnéli-B, (**e, f**) Magnéli-C, (**g, h**) TiO_2-A, (**i, j**) TiO_2-B in fully supplemented and serum-deprived cell medium. The arrow indicates the start of perfusion with NPs. For evaluation of the intracellular Ca^{2+} level, Ca^{2+}-sensitive Fluo-4 dye was used. For each treatment condition, three independent repetitions were performed where the fluorescence of at least 20 individual cells was evaluated. Results are presented as an average Fluo-4 fluorescence (+SD) according to vehicle treated control cells (dashed line). Asterisk presents significant difference with respect to the control cells (* equals $p < 0.05$; ** equals $p < 0.01$; *** equals $p < 0.001$; ANOVA with Bonferroni's post test).
